# Trees on networks: resolving statistical patterns of phylogenetic similarities among interacting proteins

**DOI:** 10.1186/1471-2105-11-470

**Published:** 2010-09-20

**Authors:** William P Kelly, Michael PH Stumpf

**Affiliations:** 1 Centre for Bioinformatics, Imperial College, London, UK; 2 Centre for Integrative Systems Biology at Imperial College (CISBIC), London, UK; 3 Institute of Mathematical Sciences, Imperial College, London, UK

## Abstract

**Background:**

Phylogenies capture the evolutionary ancestry linking extant species. Correlations and similarities among a set of species are mediated by and need to be understood in terms of the phylogenic tree. In a similar way it has been argued that biological networks also induce correlations among sets of interacting genes or their protein products.

**Results:**

We develop suitable statistical resampling schemes that can incorporate these two potential sources of correlation into a single inferential framework. To illustrate our approach we apply it to protein interaction data in yeast and investigate whether the phylogenetic trees of interacting proteins in a panel of yeast species are more similar than would be expected by chance.

**Conclusions:**

While we find only negligible evidence for such increased levels of similarities, our statistical approach allows us to resolve the previously reported contradictory results on the levels of co-evolution induced by protein-protein interactions. We conclude with a discussion as to how we may employ the statistical framework developed here in further functional and evolutionary analyses of biological networks and systems.

## 1 Background

The biological structure and function of organisms at the cellular level are the product of interactions between proteins and other molecules. The resulting networks of biological interactions found in an organism have been studied using concepts from graph theory, and the quantitative analysis of biological networks has become important for the description of biological systems [1-3]. While protein-protein interaction (PPI) data are incomplete, and in most instances suffer from either abundant noise or potential experimental bias as a consequence of prior biological knowledge [4,5], there have been numerous reports over the last decade highlighting the use of PPI network data, including how these can be used to understand molecular processes, disease phenotypes, and evolutionary properties of biological systems (*e.g*. [6,7]).

In addition to their functional role, protein interaction networks (PINs) have also been analyzed from an evolutionary perspective. A host of analyses have studied, e.g. the potential link between the evolutionary rate of a protein and its degree or position in PINs [8-16]. Similarly, properties of interacting proteins have been investigated in order to determine whether or not their properties are more similar than those of non-interacting proteins [16-18]. Such an evolutionary signature could, for example, be used to predict protein-protein interactions from comparative analyses of biological sequence data. But many of these studies have reported partially contradictory results: some analyses find a clear (but weak) correlation between degree (or other network measures such as centrality) and protein evolutionary rate, while others fail to detect any statistical signature in the data. The problem of reconciling these conflicting findings is exacerbated by a number of factors: different analyses use different protein interaction data; potentially confounding factors are not always accounted for; statistical approaches differ; and the underlying protein interaction and evolutionary data are themselves subject to considerable uncertainty and variability.

Here we develop and discuss suitable statistical frameworks for the analysis of highly structured network data. Most previous analyses have compared the observed network data with randomized networks where generally only the degree sequence was maintained by the randomly rewired networks [19,20]. Such an approach is based on the implicit (and rarely explicitly stated) assumption that the main contribution of the network to the evolutionary characteristics of the constituent proteins stems from the different number of interactions proteins have. This is a sensible and computationally convenient, yet potentially very restrictive statistical null model.

Here we apply our statistical framework to the analysis of phylogenetic trees of proteins generated for a panel of yeast species, where we can discuss the relative merits of different null models on the evolutionary analysis. In particular, we will seek to quantify the similarity of the phylogenetic trees of interacting proteins. These trees capture a different level of correlation in the overall data: the correlations between orthologous proteins in different species. High levels of concordance between the individual proteins' phylogenetic trees are anticipated as these should tend to follow the species tree [21]. However, whether or not characteristics of phylogenetic trees, especially their topology, show concordance between interacting proteins greater than would be expected by chance in random graphs has not previously been explicitly tested on a global level. We assess the extent of such similarities for different protein interaction datasets and different phylogenetic reconstruction methods in order to determine the level of variability arising from differences in the data or methodological differences alone, and mitigate for this if possible.

Section 2 introduces the different network null models that are used to compare the phylogenies under different assumptions regarding the underlying PIN. These enable subtly different questions to be asked about any correlation which is discovered between the topology and reported PPIs. The PIN data used in the study are also introduced here as well as the similarity measures used to assess linkage between interactions and phylogenetic trees. Section 3 presents the results of the topological analysis, including association between PPIs and phylogenetic profiles in yeast, and the main differences between different PIN datasets and phylogenetic tree methodologies. Section 4 draws the results together and concludes that although there is correlation between topological similarity and PPIs when compared to all protein pairs, this is not true once the network structure is constrained to reflect the structure of the empirical data. This shows the importance of using an appropriate null model when seeking significant biological traits in interactome studies and also adds to evidence from alternative sources regarding the role of co-evolution, or correlated evolutionary rates, and how this relates to the observed interactions in *S. cerevisiae*.

## 2 Methods

Here we describe the methods used to compare different phylogenetic trees for each protein found in *S. cerevisiae*. First, the sources of data are outlined. Second, we discuss how to compare the topologies of phylogenies. Finally, we describe the network ensembles, which are used to represent statistical null models for PINs.

### 2.1 Data

**Figure 1:**
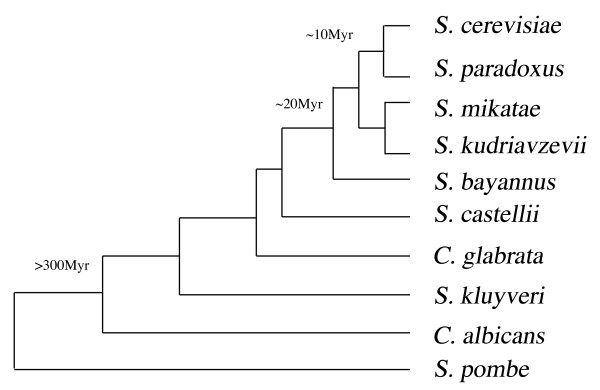
**Phylogeny of study species**. This shows the phylogenetic tree for the study species used in this paper. The evolutionary relationship is shown for the ten yeast species used [53,54]. *S. cerevisiae *proteins resulting from gene duplication events are thought to retain the same interactions as the original gene for millions of years rather than tens or hundreds of million years [55].

To generate phylogenetic trees for each *S. cerevisiae *protein, a selection of 9 other yeast species, from the *Saccharomyces *and *Candida *genera, have been mined for orthologous proteins to all *S. cerevisiae *proteins using a best-reciprocal-hit BLAST approach [15]. The 10 species are thought to have diverged from *S*. *cerevisiae *between approximately 10 million years (*S. paradoxus*) and over 300 million years (*S. pombe*) as shown in Figure 1. Multiple sequence alignments (MSA) were obtained using CLUSTALW [22] for each *S*. *cerevisiae *protein and its identified orthologues.

Three different algorithms were used to infer the protein phylogenetic trees from the MSAs: PARS and PROML from Phylip 3.6 [23]; and the Codonml routine from PAML [24]. For each inference method, the analysis is restricted to those proteins where trees were determined unambiguously (as an algorithm can return multiple trees with equal confidence). For each protein, we analyzed both gapped and ungapped sequences but did not detect any differences between the two sets; results shown here are from the gapped set.

The species tree, shown in Figure 1, and the protein trees may not necessarily agree [25]. As well as the protein trees being on a subset of the 10 study species (dependent on the availability of homologues) the topology may also be different. The evolutionary history between species is described by the species tree, whilst a protein tree represents how a set of homologous proteins may have evolved relative to each other. Such differences can become particularly apparent when the divergence time between the species is short [26], so for the yeast species used here there should be apparent variability between the trees produced.

In order to generate the random networks, an empirical PIN for *S. cerevisiae *is also necessary. As there is still uncertainty over the network (regarding both the interactions and overall size of the graph), three different datasets are used. CORE and DIP graphs are formed from the data contained in the Database of Interacting Proteins (DIP) [27], whilst a literature curated graph (LC) has been taken from BioGRID [28]. The LC dataset [29] forms a combination of small scale and high-throughput experimental results were obtained as part of a large scale literature curation exercise of the available interactome studies found in *S*. *cerevisiae*.

The three empirical graphs form different samples of *S. cerevisiae *PPIs, containing data that has been hand curated (LC), passed some expert criteria [30] (CORE), or is a complete interaction database (DIP). Table 1 details some of the graph statistics for each of the three empirical graphs.

**Table 1:** Protein interaction networks

Graph	Proteins	Interactions	Components	Maximum degree	Mean degree	Clustering coefficient
**CORE**	2,528	5,728	78	91	4.8	0.21
**DIP**	4,931	17,471	31	283	7.0	0.10
**LC**	5,109	21,283	42	319	8.5	0.13

Statistics for the PINs used to complete the phylogenetic analysis. These have been taken from BioGRID (a literature curated subset) and DIP. The LC data form the largest network dataset, whilst the CORE data are a subset of the DIP interactions which are more highly clustered but formed of a higher number of individual components.

A number of biological features have been proposed as means of classifying protein-protein interactions [31-34], or linked with PPIs [20,35,36]. A collection of these biological characteristics are used here to generate conditional random graph null models: (a) molecular function, biological process or cellular component annotations taken from the GO slim ontology [37]; (b) multi protein complexes found in [38]; and (c) mRNA expression levels as a proxy for *S. cerevisiae *protein expression levels from [39].

### 2.2 Protein phylogeny similarity

In order to explore the role of evolutionary constraints on *S. cerevisiae *protein pairs and how these may be linked to the reported *S. cerevisiae *PIN the similarity of phylogenetic trees is assessed using two different measures. First, the differences found between the phylogenetic profiles of protein pairs are used; this relies only on the orthologues identified rather than the inferred protein phylogenetic trees. Second, the similarity of the topology of the phylogenetic trees for pairs of proteins are used to assess how PINs, PPIs and the phylogenetic trees relate to one another.

#### 2.2.1 Phylogenetic profile

The orthologue information is used to construct the phylogenetic profiles for each protein [8]. Proteins for which no orthologues are available have been excluded from the analysis (the profile would be trivially the null vector and is excluded to avoid bias). The phylogenetic profiles of a protein pair are compared by counting the species in which both either exhibit, or do not exhibit, an orthologous protein. Accordingly, the score between two protein phylogenetic profiles is between 0 (available orthologues for both proteins occur in the same species) and 9 (only one of the proteins has an orthologue in each assessed species).

#### 2.2.2 Tree topology

Proteins are said to co-evolve if they have similar evolutionary paths [40] where the mutational changes in one protein are triggered by changes in the co-evolving protein -- *i.e*. the changes are compensatory. One consequence of co-evolution between protein pairs is a tendency to see similar rates of evolutionary change which are reflected through the branch lengths exhibited on the protein phylogenetic trees [41]. These branch lengths, whilst indicative of possible co-evolutionary behaviour, may also be indicative of correlated evolutionary rates, which may also be non-compensatory, as has been shown in *S. cerevisiae *[42]. The correlation observed in the evolutionary distances is a consequence of constraints on the evolutionary rate, rather than a consequence of compensatory changes.

Whilst co-evolutionary behaviour between proteins will influence their rates of evolution, it also could affect the topology of their respective phylogenetic trees. If the proteins do interact, then each divergent split (reflected in the topology) will trigger changes in the co-evolving protein. If proteins *A *and *B *co-evolve then any evolutionary change in protein A will trigger compensatory changes in the second protein *B *- and *vice versa*.

The patterns of divergence, as a proxy for possible co-evolutionary changes, are measured through recording the similarity between phylogenetic topologies. In order to assess how well these topologies reflect the co-adaption which may be present in interacting protein pairs, above that found in the protein population as a whole, the similarity between phylogenetic topologies is measured. The similarity between proteins is highly dependent on the level of protein divergence within the species (the trees are not independent observations), but should be linked with the observed PPIs if there is evidence that protein pairs co-evolve as a consequence of interactions.

**Figure 2:**
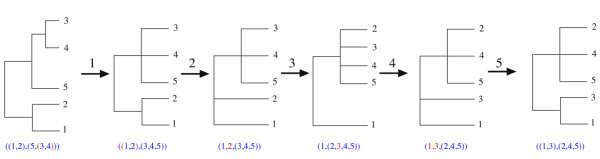
**Topology edit distance**. This shows an example of how the edit distance is found for different topologies on the same number of species (in this case 5 different lineages). Each move allows the splitting or joining of a pair of lineages. The score is then used to find the similarity between tree topologies taking into account the number of species being compared. This example compares the tree topologies: ((1,3),(2,4,5)) and ((1,2),(5,(3,4))). The score here is 5.

Given two protein trees, their topologies match if the phylogenies, on the set of species that appear in both topologies, are non-trivially identical. This requires that the two trees share at least 3 distinct species. If they do not match on the set of shared species, then to measure the similarity of the two trees, an edit distance, *η*, on a set of *n *species can be defined. This distance is based on a nearest-neighbour interchange method [21]. Each phylogenetic topology, *e.g*. ((1, 2), (5, (3, 4))), contains a set of species, {1,2,3,4,5}, and divergence events or internal nodes where lineages split (here represented by brackets). Topologies are neighbours of one another if they can be made identical by moving a single species across a node. For the string tree notation, *across a node *means either: (i) swapping a species with the first *bracket *either side in the string (and deleting unnecessary brackets); or creating a *bracket *around two species in the same set of *brackets *- *e.g*. (1, 2, 3) is a neighbour of ((1, 2), 3). For example, for ((1, 2), (5, (3, 4))) the neighbours are: (1, 2, (5, (3, 4))), ((1, 2), (5, 3, 4)), and (5, (1, 2), (3, 4)). Figure 2 shows a minimal sequence of neighbouring phylogenetic trees to travel from topology ((1, 3), (2, 4, 5)), for protein *A*, to ((1, 2), (5, (3, 4))), for protein *B*. The distance, *η_A, B_*, is the minimum number of tree topology changes required to generate matching trees.

Each protein may have a different number of homologous proteins on which the phylogenetic tree is based. The edit distance is thus not directly comparable between protein pairs as the possible number of trees is dependent on the number of species found in each tree. Consequently, let the similarity of topologies, Γ_*A, B *_∈ [0, 1], be defined as:

(1) $$\begin{array}{l} \Gamma_{A,B}=1-\frac{\eta_{A,B}}{M_{n}}. \end{array}$$

where *η_A, B _*is the score between two trees sharing the same *n *species and *M_n _*is the maximum possible score between two trees on *n *species. The maximum edit distance between two phylogenetic trees on *n *species is found by the recursion:

(2) $$\begin{array}{l} M_{n+1}=M_{n}+\left(n-2\right), \end{array}$$

with *M*_3 _= 2.

For rooted bifurcating trees the total number of possible topologies for *n *species, *T_n _*is:

(3) $$\begin{array}{l} T_{n}=\frac{\left(2n-3\right)!}{2^{n-1}\left(n-1\right)!} \end{array}$$

Multifurcating rooted trees may have more than two lineages at each internal tree node, so the number of different topologies on *n *species is larger than bifurcating trees. The number of trees is given by the sum over the number of internal nodes, *m*, found in the tree:

(4) $$T_{n,m}=\left(n+m-2\right)T_{n-1,m-1}+T_{n-1,m},$$

for *m *∈ [1, *n *- 1], *T*_*n*, 1 _= 1 and *T_n, m _*= 0 ∀ *m *≥ *n*. The possible topologies, and associated maximum scores, *M_n_*, between distinct trees on *n *species are shown in Table 2.

**Table 2:** Number of topologies

Species	Bifurcating trees	Multifurcating trees	Maximum score
1	1	1	-
2	1	1	-
3	3	4	2
4	15	26	4
5	105	236	7
6	945	2, 752	11
7	10, 395	39, 208	16
8	135, 135	660, 032	22
9	2, 027, 025	12, 818, 912	29
10	34, 459, 425	282, 137, 824	37

The number of rooted labelled trees for *n *species. This shows the how the number of possible topologies is related to the number of shared lineages being compared in each protein tree comparison.

### 2.3 Null networks

A variety of different graph ensembles, or *null models*, have been used for PIN analyses [13,19], although the rationale for their choice is not always clear. Assumptions regarding how the graph is structured or about its size and order may bias conclusions, leading to a model that is not appropriate for our hypothesis, and risk falsely dismissing findings or generating false positive conclusions [43]. In practice, it is difficult to find a truly null model for the generation of complex, correlated (and contingent) data [44,45]. When generating random graphs (where the properties of the nodes and edges are important) it can be hard to define a satisfactory parameter set that should be fixed for comparison with observed data. In order to assess the possible dependence between some protein characteristic (such as GO annotations) and PPIs, or the PIN structure, the data need to be assessed across different random graph null models as defined in this section. Whilst these null models can be used for any statistical test, here the characteristic of interest in this study is the similarity of protein phylogenetic tree topology.

A graph or interaction network, *G *~ (*V_G _*, *E_G_*), has a set of proteins *v_i _*∈ *V_G _*and interactions between pairs of proteins, (*v_i, _v_j_*) ∈ *E_G _*(self-interactions are not considered here, hence *i *≠ *j*). Let the order *n *be the number of proteins found in *V_G_*, and the graph size *m *be the number of edges found in *E_G_*. The null models generate a random graph, *H *~ (*V_H_*, *E_H_*), with the same order (in fact, the same protein set) and size as the observed network, *G*. The proteins also have a collection of biological traits, *β*(*v_i_*), such as evolutionary rate, and topological traits, *ϕ*(*v_i_*), such as the degree, which may be used to generate conditional random graphs from the observed data.

Graph rewiring [46] is used to generate random graphs from empirical data where each rewiring maintains both the size, *n*, and order, *m*, of the empirical graph used. An edge, *e*, is rewired if it is deleted from the graph edge set, *E*, and a new edge, *e'*, is added to the graph from the same node set, *V*. A graph, *H *~ (*V_H_*, *E_H_*), is a rewiring of *G *~ (*V_G_*, *E_G_*) if |*E_H_*| = |*E_G_*| and *V_H _*= *V_G_*.

Each random graph considered is a sample from a graph ensemble (forming a particular probability distribution over the space of graphs with *n *nodes and *m *edges). Comparisons are made between the empirical graph and those found when sampled from the graph ensemble. Consequently, the ensemble serves as a null model for the analyses presented here. The empirical graph is referred to as *G *throughout.

#### 2.3.1 Topological ensembles

**Figure 3:**
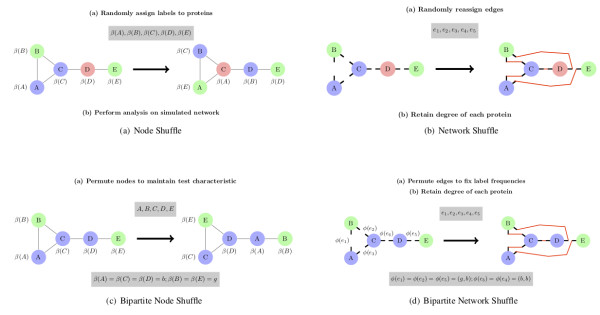
**Graph ensemble null models**. The graph null models are described in the four figures shown here. Each graph is generated from a fixed empirical graph (CORE, DIP or LC) according to one of the four different algorithms. (a) and (b) permute purely network structural information while (c) and (d) constrain the generation of random graphs also according to biological constraints (such as GO ontology information and complex annotations). **(a) Node shuffle: **The labels for each node are permuted (*e.g*. node colour) but the topology of the graph is fixed. **(b) Network shuffle: **The degree of each node, [A, B, C, D, E], is fixed along with the node characteristic, colour, whilst the edges are randomly rewired. **(c) Bipartite Node Shuffle: **This permutes each node to another node, *v_i _*→ *v_j _*such that *β*(*v_i_*) = *β*(*v_j_*) for the particular characteristic, *β*, under consideration. **(d) Bipartite Network Shuffle: **This retains the degree of each node, *d_G_*(*v*), and also rewires each edge, *e*, to one of the available node pairs that share the same edge characteristic, *ϕ*(*e*).

Three different graph ensembles (shown in Figure 3) are used that fix certain network traits of the empirical data: *random graph*; *node shuffle*; and *network shuffle*. These take account of the degree sequence, size, and order of the empirical graph. However, these null models take no account of any biological data or knowledge in order to generate random graphs, solely replicating topological traits of the empirical PINs.

##### Random graph

A graph, *H*, from this ensemble is generated using the Erdös-Rényi (ER) graph model [47]. Throughout this paper an ER graph is defined as a graph, *H *(*n*, *m*), with *n *nodes and *m *edges placed uniformly and at random across all possible edges, (*v_i_*, *v_j_*) where *i *≠ *j*. Accordingly, this model produces a graph, *H*, of identical order, *n*, and size, *m *as the original graph *G*. Biological node traits (such as sequence or annotations) are fixed and the *m *edges are sampled uniformly without replacement.

##### Node shuffle

A graph sampled from this graph ensemble retains all network traits of the empirical graph, maintaining the adjacency matrix, *A*. The node traits are permuted amongst all the nodes of the graph, *G*. Although the generated graph, *H*, has identical structure to the empirical graph, the node traits, *β_G_*(*v*), are randomly allocated amongst the nodes, *V*. This enables assessment of whether the node characteristics depend on the structure of the graph.

##### Network shuffle

This graph ensemble generates graphs that preserve network traits, using the rewiring algorithm presented by [46]. The degree of each node, *d_G_*(*v*), along with each node biological trait, *β_G_*(*v*), are fixed, and edges are randomly distributed under these constraints. The number of legal moves may be small under certain conditions, primarily as the proportion of possible edges increases. In the case of PIN data this is not a concern in general as the graphs are sparse:

(5) $$H\sim (V,E^{\prime })\text{ where for each }v_{i}\in V,d_{H}(v_{i})=d_{G}(v_{i}).$$

#### 2.3.2 Biological ensembles

The following graph ensembles (shown in Figure 3) fix both network and biological traits of the empirical data: *biological node shuffle *and *biological network shuffle*. The generated graphs retain some of the biological properties, such as interactions between proteins found in the same complex or those with the same functional GO annotations, as well as possibly the topological properties as found for the topological ensembles.

The characteristic, *β*, used in each of these examples can be any node, or edge, property. For this study, the graph ensembles are generated for the following selection of different characteristics: GO slim [37] annotations for biological process **[process]**, cellular component **[component]**, and molecular function **[function]**; and multi-protein complex annotations **[complex] **found in [38]. These different ensembles produce random graphs which constrain different biological elements of the graph, allowing us to evaluate whether these form confounding factors that may explain the observed phylogenetic properties.

##### Biological node shuffle

This ensemble permutes the nodes such that each node, *v_i_*, is switched with one, *v_j_*, sharing a particular characteristic, *β*(*v_i_*) = *β*(*v_j_*), as shown in Figure 3(c). Each graph can be thought of as being generated in the same means as *node shuffle *graphs although there is an extra biological constraint on how each of the proteins are permuted.

##### Biological network shuffle

This graph ensemble is based on the algorithm used to produce the *network shuffle *graph ensemble, and shown in Figure 3(d). An edge, *e_h_*, has a characteristic, *ϕ*(*e_h_*), determined by characteristics of the nodes it connects, *ϕ*(*e_h_*) = *ϕ*(*v_i_*, *v_j_*). Each edge is rewired to maintain the degree of each node, *d_G_*(*v*), as in *network shuffle*, and retains the characteristic of the rewired edge, *e_h_*. So *e_h _*→ *e'*_*h *_if *ϕ*(*e_h_*) = *ϕ*(*e'_h_*).

Graphs are sampled uniformly from each ensemble. The similarity, Γ, score, *η*, and difference between the phylogenetic profiles are measured in order to assess differences between the putative PPIs reported in the observed PINs - CORE, DIP, and LC - and random graphs sampled from the random null models. For the analyses, 1,000 graphs are sampled from each graph ensemble.

For each graph, the topological results are only recorded if a valid tree comparison can be made. There are two sources of unknown data for the phylogenetic comparisons: (i) there is no known tree data for a considered protein; or (ii) the protein trees share fewer than 3 lineages so a topological comparison is unable to be completed. Both sources of variation in the overall number of comparisons made are recorded for each ensemble to assess how they may potentially bias the results. In order to avoid biasing the results, if the trees do not share 3 lineages they are ignored from the following analyses, although the number of comparisons made in each case is also recorded to assess whether it affects the overall outcome alongside the number of lineages compared in each analysis.

## 3 Results

Phylogenetic profiles are assessed for each empirical graph and ensemble method. This is followed by an analysis of the topological similarity of interacting proteins and finally by a comparison of the three different phylogenetic tree construction algorithms. The results presented in this section focus on the PROML phylogenetic trees (as the trends for phylogenetic topology similarity across the methods used were similar), although there is also a comparison for the results from the three different phylogeny techniques used. The number of trees generated (owing to either no result from the algorithm or ambiguous trees) for the methods are: PROML - 4,380; PARS - 3,617; and PAML - 4,260.

Our network ensembles probe different aspects of a putative association between the PIN and phylogenetic properties of the constituent proteins. *Node shuffle *graph ensembles fix the graph structure and the phylogenetic tree labels are permuted randomly amongst the nodes. *Network shuffle *graph ensembles associate a tree phylogeny and degree with each node but randomise the interactions. These probe the relative similarity of interacting phylogenies in differently organized random graph.

### 3.1 Phylogenetic profiles

**Figure 4:**
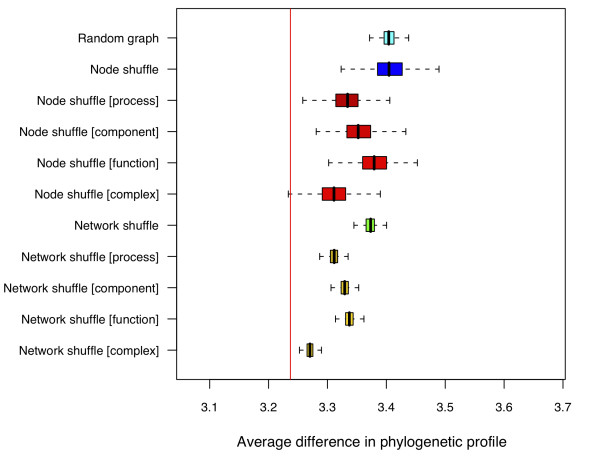
**Phylogenetic profiles for each ensemble**. Boxplots of the phylogenetic profile differences for edges across the assessed null model ensembles. The red line shows the average phylogenetic profile difference found for edges found in the LC graph. The observed phylogenetic difference found between PPIs in LC is significantly less than seen in the graphs sampled from each random null model, although the *network shuffle *graphs exhibit lower differences than the *random graph *ensemble graphs. The results are also shown for ensembles constrained using the GO and complex annotations.

Figure 4 shows the phylogenetic profile differences found for the sampled ensembles together with a red line showing the average for the LC data. The proteins have on average more than five identifiable orthologous proteins across the nine study species. Across all the ensembles, the phylogenetic profile difference is higher in general for the graphs sampled from each ensemble than in the empirical data. *Network shuffle *ensembles on average produce a closer trait to the LC graph than the *node shuffle *ensembles. Similarly, the *node shuffle *ensembles has higher variability than either the *random graph *ensembles or the *network shuffle *ensembles. If the edge rewirings are constrained by complex annotations (the **[complex] **ensembles) the phylogenetic profile differences are most similar to those found in the empirical graph.

**Figure 5:**
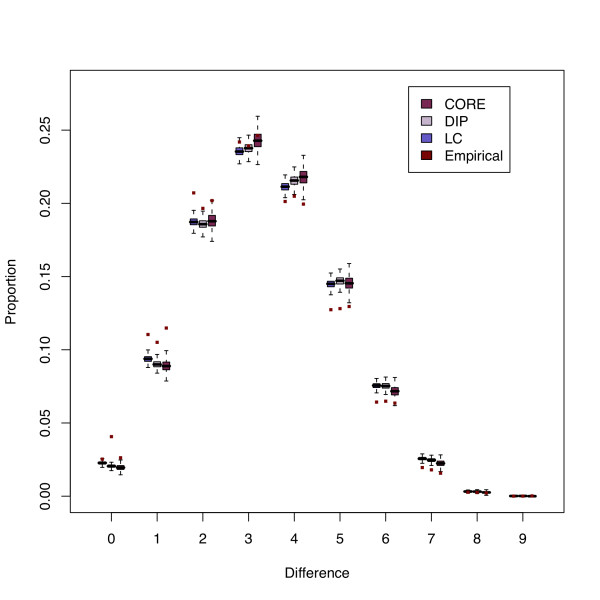
**Phylogenetic profile differences**. This figure presents the phylogenetic profile differences found for the random graphs generated based on the 3 different empirical datasets in comparison to those found in the original graph. The red points show the proportion found in the empirical PIN dataset, whilst the boxplots present the data from the random graphs. The empirical data, shown as red dots for each boxplot, are generally higher for differences less than 4, showing that the observed PPIs are more likely to share phylogenetic profiles than those edges found in any of the random graph ensembles.

Figure 5 compares the true number of differences for each of the empirical graphs with the *random graph *ensemble graphs. The differences observed here are matched across the other graph ensembles and can be seen more fully in the Additional File 1. The proportion of interacting proteins (the red dot indicates the observed data) are shown for each possible phylogenetic profile (0-9). The horizontal axis shows the differences between phylogenetic profiles. For all graph datasets, and across all different graph ensembles (see in Additional File 1), the phylogenetic profiles with three or fewer differences are found more often among the real interacting pairs than in tested random graph ensembles. Although the graphs are of different sizes, the PPIs in each of them show similar phylogenetic profile differences in both the random ensembles and empirical data. An exception to this is the DIP graph, where a higher proportion of edges are found between proteins that have matching phylogenetic profiles.

The absence and presence of orthologues in the phylogenetic profiles was also compared between interacting proteins and those generated for random graphs. The Jaccard distance for both the absence and presence of orthologues is significantly higher in the empirical data than found in the randomly generated graphs. For LC the Jaccard distance for the presence of shared lineages was found to be 0.615 whilst it was on average 0.0604 in the *network shuffle *and random graphs. The absence of shared lineages in the true PPIs data was found also to be significantly higher than found in the random ensembles, thus reinforcing the results from the phylogenetic difference measures.

### 3.2 Topological similarity

Topological similarity of a pair of protein trees is measured in three ways: the proportion of matching topologies; the topology score, *η*; and the similarity score, Γ. The similarity found in the empirical data should be higher than that for random ensembles if there is any evidence for co-evolutionary behaviour among pairs of interacting proteins. Here we describe results for the PROML phylogenetic trees as the overall behaviour is identical for each of the tree construction methods (PAML, PROML and PARS).

In each case, a number of protein tree comparisons cannot be analysed as either protein does not have the necessary orthologous information to generate an unambiguous phylogenetic tree. Across the random graphs this means that a varying proportion of potential comparisons are missed which is in general only slightly higher than the number of unknown comparisons for the empirical data. A lack of phylogenetic protein tree information results in between 10% and 25% of the comparisons being excluded, although the *network shuffle *graphs result in a similar number of excluded comparison to those seen for the empirical graph data by construction (see Additional File 1).

The number of shared species can potentially bias results. Although there is variation across the ensembles, the *network shuffle *graphs results are not significantly affected by this bias (see Additional File 1).

**Figure 6:**
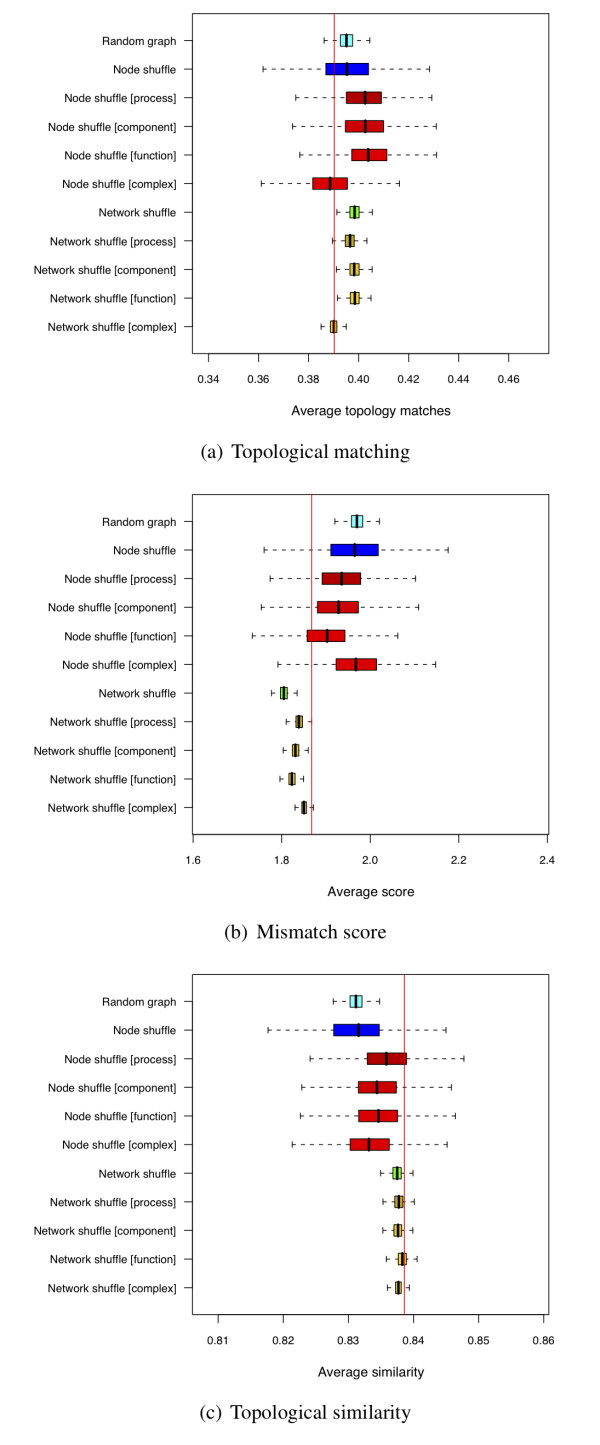
**Phylogentic topology similarity measure results**. **(a) Topological matching for LC interaction graph: **Boxplots for the distribution of matching topologies found for each sampled graph ensemble. The red line shows the result for the LC graph (also in (b) and (c)). The **[complex] **constrained graphs are the only ensembles that present fewer matching topologies than are found in the empirical data. **(b) Mismatch score using LC interaction graph: ***Node shuffle *and *random graph *ensembles exhibit higher scores than found in the empirical data, whilst *network shuffle *ensembles under any of the tested constraints exhibit a lower average score than is found in the empirical graph. **(c) Topological similarity for LC interaction graph: **The empirical data have similar similarity values as those found for the *network shuffle *graphs, whilst the similarity is significantly higher for each of these than is found in graphs sampled from the *random graph *ensemble. For each plot, the results are shown for the simple topological ensembles and also those graph ensembles which are constrained using biological traits. The three different plots highlight the sensitivity of the results to the metric used, as well as the different interpretations that may be found given different null graph models used for comparison.

Figure 6(a) shows the proportion of matching topologies for each of the graph ensembles compared to the proportion found for the LC graph (shown as a red line). Once again, the *node shuffle *ensemble shows higher variance of the trait. Each of the *biological node shuffle *ensembles constrained by a GO category exhibits a higher proportion of matching topologies than either the LC or any of the *network shuffle *or *random graph *ensembles. Indeed, the average level of topology matching seen in all but the **[complex] **constrained ensembles is higher than in the LC graph.

Figure 6(b) shows the topological mismatch scores between interacting protein pairs in LC and the graph ensembles. The topological score, which measures the average score between phylogenetic topologies, is higher for the *random graph *and *node shuffle *ensembles than for LC data. Thus interacting proteins have more similar trees, if measured by the average score, than would be expected from either the *random graph *or *node shuffle *ensembles. However, the average mismatch score for the *network shuffle *ensembles is even lower than seen for the LC graph. Figure 6(b), however, should be treated with caution as it is not normalised for the effect of comparing trees on differing numbers of species (or lineages). The similarity score is used to counter this factor, and reveals that this counter intuitive result may solely be a consequence of the influence of the number of lineages compared. The smaller average score found for the *network shuffle *ensembles needs to be reconciled with the slightly smaller number of shared orthologues found in the data (see Additional File 1).

Figure 6(c) shows the same results for the similarity measure, Γ, which takes account of the number of lineages and the score when comparing phylogenetic topologies. Unlike the results for the average score, all of the *network shuffle *ensembles are now in agreement with the empirical graph. *Node shuffle *ensembles have a lower similarity measure than the empirical data, although the sampled distribution of average similarities overlaps with the empirical result.

The variance observed from the *node shuffle *results is caused by the role of the highly connected proteins in the network data. However, it should be noted that these hubs do not have a significantly different level of similarity to the species tree, or other protein trees than less connected proteins (see Additional File 1). The average number of orthologues found for the 12 most connected proteins is marginally lower than the level for the complete set of proteins.

### 3.3 Phylogenetic methods

Table 3 shows the similarity trait, Γ, for each of the empirical graphs using trees constructed by each of PAML, PARS and PROML. There are differences between the trait statistics for each of phylogenetic construction algorithm, but the trends are similar when comparing the traits produced by each graph ensemble with those seen empirically. Differences between the phylogenetic algorithms are reflected in the level of matching topologies found in the empirical data for each tree algorithm. For example, in the case of the CORE data, phylogenies inferred using PAML match in approximately 17% of comparisons; phylogenies inferred using PROML match 42%; and phylogenies inferred using PARS match in 57%. The contrasting results for these phylogenetic algorithms are to a large extent explained by the differences between the use of *bifurcating *and *multifurcating *topologies by the construction methods and how this influences the number of possible trees [21].

**Table 3:** Similarity for each phylogenetic tree construction algorithm

Tree construction	Graph	Real	Similarity, Γ			
			*Node shuffle*		*Network shuffle*	
PAML	CORE	0.74	0.747	[0.737,0.757]	0.742	[0.737,0.747]
	DIP	0.75	0.760	[0.750,0.769]	0.743	[0.740,0.746]
	LC	0.74	0.750	[0.741,0.760]	0.741	[0.739,0.743]

PROML	CORE	0.84	0.848	[0.839,0.857]	0.846	[0.843,0.849]
	DIP	0.84	0.841	[0.834,0.849]	0.838	[0.837,0.840]
	LC	0.84	0.831	[0.822,0.839]	0.837	[0.836,0.839]

PARS	CORE	0.90	0.901	[0.893,0.909]	0.899	[0.896,0.903]
	DIP	0.90	0.893	[0.884,0.900]	0.893	[0.891,0.895]
	LC	0.89	0.882	[0.874,0.890]	0.893	[0.891,0.895]

Average similarity, Γ, for each phylogenetic tree construction algorithm, for the empirical graphs. The results for *node shuffle *and *network shuffle *graph ensembles are given along with the 95% sample range for the similarity trait.

**Figure 7:**
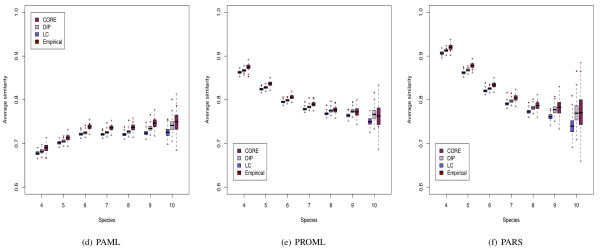
**Similarity of topologies for different tree algorithms**. Average level of similarity for topology comparisons (when rewiring using *network shuffle*) made for a fixed number of shared species. The average similarity for each method is different, and the trends seen in PAML show marked difference to those methods taken from Phylip. The variance of similarities for each tree construction method increases as more species are compared. This may potentially bias the score results for the topological comparisons, thus suggesting the need to be vigilant when comparing graph ensemble results with empirical data. The *network shuffle *results exhibit a distribution of comparisons over the species similar enough for this to not affect the overall conclusions (see Additional File 1).

Figure 7 shows the average similarity for comparisons made on a fixed number of orthologues for each of the tree construction methods. Figure 7(a) shows results obtained from PAML phylogenetic trees we see that the similarity of phylogenetic topologies increases with the number of shared orthologues. These similarity levels range from 0.65 to 0.80 for the empirical PIN data. For the other tree inference procedures (PARS and PROML), however, we observe the opposite results that the topology similarity decreases considerably as the number of shared orthologous sequences increases.

## 4 Discussion

This paper highlights the importance of using appropriate null models when testing hypotheses on large scale graphs. The biological trait, in our case the phylogenetic similarity of proteins, under consideration should be tested against a variety of graph ensembles to effectively dissociate the possible effects of topological, as well as other possibly biological, confounding factors from the analysis. Using different ensembles allows us to assess what 'expected by chance' means in the network context. Whereas the linkage of individual PPIs to particular traits can be made by assessment against an ER random graph, this is not true if the trait is believed to be linked to the network structure or other possibly biological covariates. The ensembles enable a more subtle view of the connection between traits of proteins and the networks structures that their interactions form, allowing us to develop tests that tell us about the significance of observations in interactomic data.

For our particular problem at hand *network shuffle *and *node shuffle *show contrasting results regarding the similarity of tree topologies. *Node shuffle *graph results suggest a marginally higher level of both topological matches, and of the similarity of empirical data. In contrast, *network shuffle *graph ensembles tend to exhibit levels of similarity that are not significantly different from those seen in the empirical data. These results are significantly different from those observed for random protein pairs (shown in the ER random graph ensemble). This highlights the importance of choosing an appropriate graph ensemble when assessing traits of biological networks.

The potential role of hubs, for example those proteins with a high degree, emerges from a comparison of the *network shuffle *and *node shuffle *ensembles. There is a far larger variability in the phylogenetic results when sampling from a *node shuffle *ensemble. This variation is primarily due to changes in the phylogenetic profile of the highly connected proteins. In *network shuffle *the topology-degree relationship remains fixed, and because degree-degree correlations are low, less variability is observed in the probability of matches of phylogenies. Furthermore, the *biological network shuffle *ensemble exemplifies how biological constraints can be used to produce graphs which closely agree with the observed phylogenetic topologies observed in the empirical PINs (although caution should be used to avoid overfitting).

We have shown that there is no significant evidence for phylogenies of interacting proteins to show higher levels of topological similarity than expected by chance in a PIN. The ndings have been confirmed by: (i) employing different phylogenetic inference approaches; (ii) using a range of different PIN data sets.

The topological similarity results contrast with the associations found between PPIs and phylogenetic profiles. In the case of these profiles, the linkages between sharing orthologous proteins and the existence of an enriched number of interactions is clear and has been well documented. Similarly, the linkage between PPIs and distance matrix metrics has also been used to justify the role of co-evolution among interacting partners. The topological similarity results here suggest perhaps that these effects are at a global level, where groups of proteins are conserved across species, rather than directly between binding partners. The role of co-expression may explain these affects, rather than co-evolutionary factors which should be noticeable when measuring the topological similarity of protein trees. Phylogenetic profiles and co-expression are better predictors of PPIs than the protein phylogenetic trees.

## 5 Conclusion

Our overall results concerning the topology of interacting proteins do not necessarily contradict previous work regarding the co-evolution of interacting proteins [10,16,48,49]. Measures of the evolutionary rate or functional similarity are not accounted for in this analysis and could be linked with interactions; in yeast (and also in *Caenorhabditis elegans*), however, there is evidence that such a correlation among the evolutionary rates on interacting proteins is at best weak [15]. Several authors have also shown that it is in fact the expression level of a gene (or a measure that may act as a proxy for gene expression level, such as the codon-adaptation index [50]) which explains most of the variation in protein evolutionary rate [13,15,42,51] and not properties related to the topology of the interaction network. This also appears to be independent of noise in, and incompleteness of, the PIN data [5]. These results add to the belief that the observed evolutionary linkage between PPIs is a consequence of evolutionary rate, as opposed to shared compensatory changes [42].

The results, however, should only be viewed as further complementary evidence, rather than a conclusive statement about the role of co-evolution between interacting proteins. The results, as with previous studies, can only be interpreted on the basis of the number of organisms considered and the available interaction data assessed. However, the results are consistent across the interactomes and phylogenetic methods, and highlight how an evolutionary signal can be very sensitive to the null model used.

Overall, graph ensembles offer a means of generating different random graph structures for network analysis. Given our general uncertainty as to the quality of protein interaction data it is not surprising that different datasets (but also different ensembles) yield different results as to how similar phylogenies of interacting proteins are [52]. Quite generally, the types of data that we have been considering here are such that inference will only be robust and reliable if potentially confounding factors are accounted for. The ensembles that we have discussed here allow us to capture such factors; and by comparing the results from different ensembles we may gain increased insights into biological systems and confidence into our findings.

## Authors' contributions

WPK performed algorithm development, data analysis and drafted the manuscript. MPHS and WPK conceived and conducted the study together, and drafted the manuscript. Both authors read and approved the final manuscript.

## Supplementary Material

Additional file 1 **Supplementary Methods and Figures**. PDF file containing a detailed analysis of factors influencing comparisons of phylogenetic profiles and phylogenies. Click here for file
