# Supplementary material for: Trees on networks: resolving statistical patterns of phylogenetic similarities among interacting proteins
Source: BMC Bioinformatics. 2010 Sep 20;11:470. doi: 10.1186/1471-2105-11-470 (PMC2955699; doi:10.1186/1471-2105-11-470)
Supplement: Additional file 1 — Supplementary Methods and Figures. PDF file containing a detailed analysis of factors influencing comparisons of phylogenetic profiles and phylogenies. [file 1471-2105-11-470-S1.PDF]

Trees on networks: resolving statistical patterns of phylogenetic similarities among interacting proteins – supplementary material

W.P. Kelly[1]and M.P.H. Stumpf*[1,2]

[1]Centre for Bioinformatics, Imperial College, London
[2]Institute of Mathematical Sciences, Imperial College, London

Email: W.P. Kelly - william.kelly04@imperial.ac.uk; M.P.H. Stumpf*- m.stumpf@imperial.ac.uk;

*Corresponding author

Phylogenetic profiles

Figure S1 shows a selection of the graph ensembles – *node shuffle*, *network shuffle*, *random graph* and *biological node shuffle* [**complex**] – against the true output for the three empirical graphs: CORE, DIP and LC. The proportion of interacting proteins (after rewiring or the red dot for empirical data) are shown for each possible phylogenetic profile (0–9). The horizontal axis shows the differences found between phylogenetic profiles, ranging from 0 (both proteins have orthologues in exactly the same species) to 9 (one of the compared proteins has orthologues in only those species that the other does not).

The results show that empirical interactions exhibit a higher propensity for similar phylogenetic profiles across all four shown ensembles. *Biological node shuffle* [**complex**] ensemble graphs are closer to the empirical data than any of the other ensembles. For all graph datasets the phylogenetic profiles with 3 or fewer differences are found more often among the real interacting pairs than in tested random graph ensembles.

Figure S1 shows that there is little difference between the results found for each empirical graph. Although the graphs are of different sizes, the PPIs in each of them show similar phylogenetic profile differences in both the random ensembles and empirical data. An exception to this is the DIP graph, where a higher proportion of edges are found between proteins that have matching phylogenetic profiles. The proportion of matching phylogenetic profiles is also higher in the *node shuffle* ensemble graphs sampled using DIP than in either the empirical results for CORE or LC or the other graph ensembles using DIP.

Phylogenetic lineages compared

Figure S2 shows the number of lineages compared, on average, for each graph ensemble technique assessed. The number of lineages compared has a knock on affect on the average scores found for each graph, and thus is required alongside the score data for a fair comparison. These data are normalised, to take affect of the number of lineages, in order to produce the final similarity score. The number of lineages compared is slightly lower for the *network shuffle* ensembles, whilst it is higher for some of the *node shuffle* and random graph ensemble instances. These results show that the *network shuffle* graphs have a tendency to match proteins with marginally less matching orthologous information whilst the other ensembles exhibit a similar number of orthologous data.

Phylogenetic topology comparisons

Unknown phylogenetic trees

There are two reasons why a given protein pair may yield no result when investigating the phylogenetic similarity. Firstly, the phylogenetic algorithms may not be able to form a protein tree owing to a lack of orthologous protein information. Figure S3 shows the proportion of topological comparisons which do not yield a result as a consequence of unknown phylogenetic information. The results shown are for each of the ensembles methods, using the LC network data and PROML phylogenetic trees. The number of unknown comparisons is larger in the random graphs than found in the empirical data. It ranges from being at the same level as found in the empirical data, for each of the *network shuffle* ensembles, to having more than twice as many unknown comparisons (averaging around 22%) for the random and node shuffle graphs. The amount of unknown data increases when the degree of each protein is allowed to change but by construction stays almost constant for *network shuffle* graphs when the structure is fixed relative to the protein tree information.

Sharing too few lineages

A second reason that two proteins may not give a topological result is if the overlap of their phylogenetic trees results in fewer than 3 species. These results do not contribute to the final analysis as they would match trivially and bias the results. Each topology comparison is made between two protein trees which have different orthologous information. Accordingly, the topological information may only overlap on a subset of the study species. At the same time, if there are fewer shared orthologues in the compared trees, it may be that there is a bias towards presenting a higher level of similarity between the phylogenetic trees.

In order to assess this potential bias, Figure S4 shows the proportion of comparisons which are made according to the number of shared lineages. It shows the empirical proportions for each of the 3 PIN datasets, and boxplots showing the levels seen for *network shuffle* and *node shuffle* random graphs, using the complex data to constrain how the random graphs are generated.

Figure S4 provide evidence that the scores seen in the randomly generated data are not being overly biased by an enriched level of comparisons made on 3 species. Indeed, for the complex data, there are on average less comparisons made on the lowest possible number of species than are found from the empirical data. Overall, the average level of lineages is higher owing to a very small number of comparisons on almost the complete set of species in the true data, rather than a large bias to the simplest possible topologies in the randomly generated ensembles.

High degree proteins and phylogenetic similarity

The variance seen in the *node shuffle* ensemble results is primarily caused by the role of high connected (hub) proteins in the LC data. Permuting the phylogenetic tree found for the highly connected proteins significantly affects the overall similarity found between these random graphs and the original empirical data. This further highlights the key role these proteins play in interactome data studies. In order to gain a better appreciation of how these proteins affect the overall analysis, the similarity of the protein trees is assessed by the degree each protein in the PROML dataset.

Figure S5 shows the level of similarity between protein trees when binned according to the degree seen in the LC dataset. It can be seen that there is no significant change in the overall level of similarity as the degree of the protein changes. Accordingly, whilst the ability to alter the hubs in the random ensembles generates a high variance in the similarity scores, it is not an overriding cause of the high levels of similarity seen in the empirical network datasets.

Figures

Figure S1 - Phylogenetic profile differences

The differences in phylogenetic profiles, for each edge, shown as a proportion of the comparisons made across the data for four different graph ensembles. The empirical data, shown as red dots for each boxplot, are generally higher for differences less than 4, showing that the observed PPIs are more likely to share phylogenetic profiles than those edges found in any of the random graph ensembles.

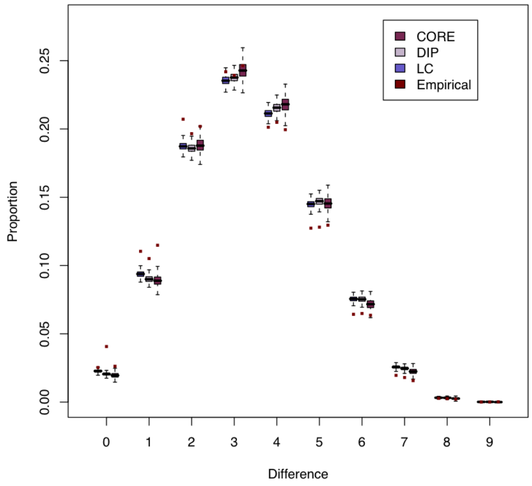

(a) *Random graph*

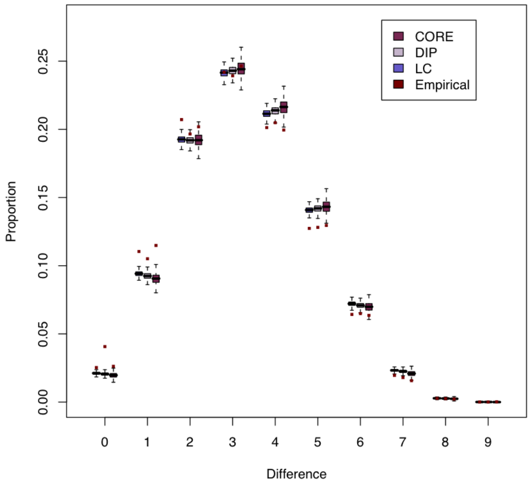

(b) *Network shuffle*

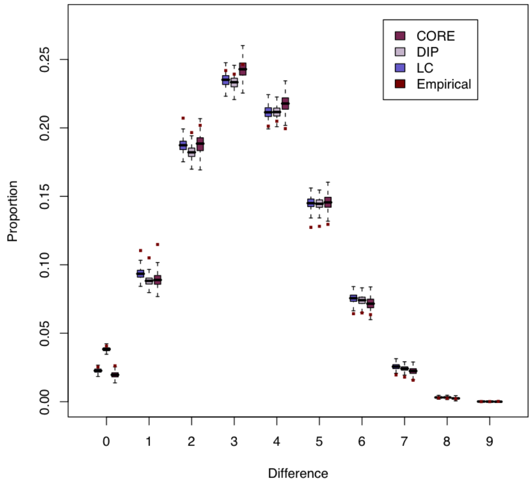

(c) *Node shuffle*

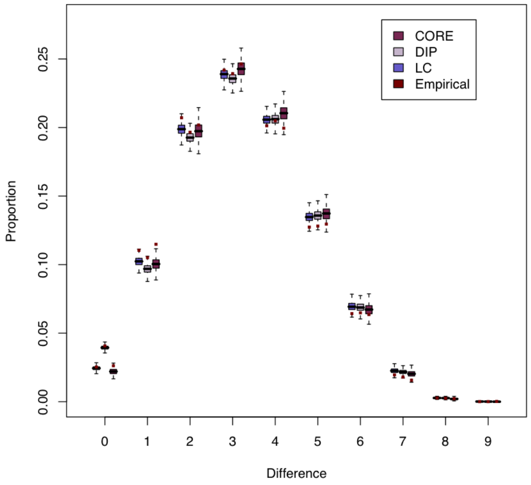

(d) *Biological node shuffle* **[complex]**

Figure S1: **Phylogenetic profile differences**

Figure S2 - Compared lineages for graph ensembles

This boxplot shows the average number of lineages compared for each graph when using the LC network data and PROML phylogenetic trees. The red line exhibits the number, on average, compared when using the empirical data, whilst the boxplots show the data produced for each of the random graph ensembles. These show that it is necessary to normalise the scores found between topologies in order to eliminate the confounding factor of shared species from the analysis.

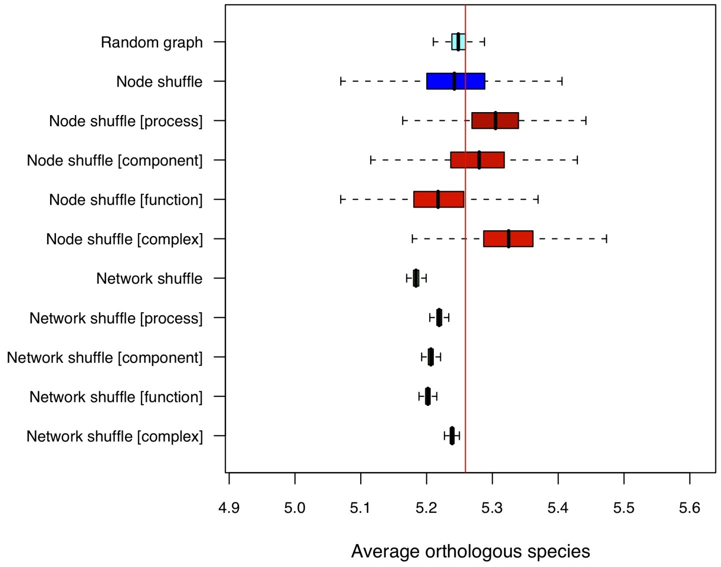

Figure S2: **Compared lineages for graph ensembles**

Figure S3 - Unknown topological comparison data

The proportion of comparisons which cannot be used to find topological similarity scores between different protein trees. The red line shows the proportion of protein pairs which cannot be compared in the LC graph data, whilst the boxplots show the range of results for the random ensemble data. Whilst the random graphs and *node shuffle* ensembles exhibit a wide variety of unknown comparisons, the *network shuffle* ensembles exhibit a consistent level close to that seen in the empirical data.

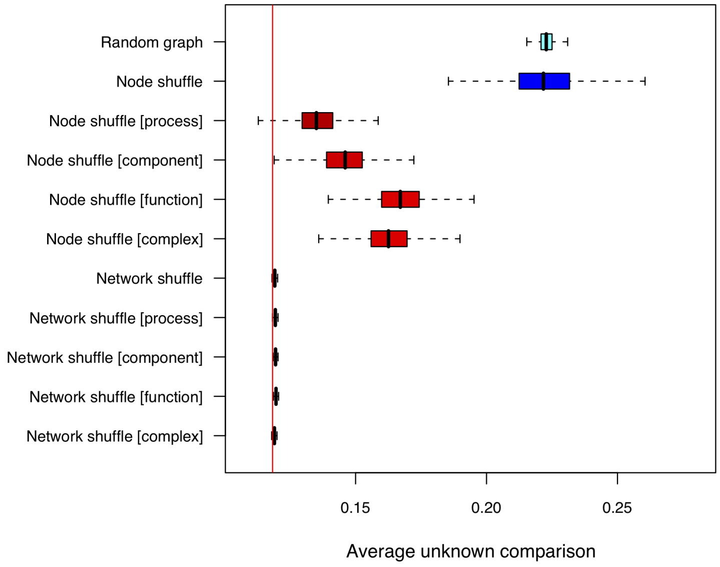

Figure S3: **Unknown topological comparison data**

Figure S4 - Matching orthologues for each topology comparison

These graphs show the proportion of comparisons made for each number of possible matching orthologues. The red dot shows the proportion of comparisons found for the empirical data, whilst the boxplots detail the proportions found when the random graphs are generated using CORE, DIP or LC as the basis graph for the ensembles. A comparison is only recorded if the species share 3 orthologues. Whilst the average number of species compared is lower in the random ensembles, this is due to a small number of comparisons made on 8 or 9 species in the empirical data, rather than a large bias for fewer species in the random replicates.

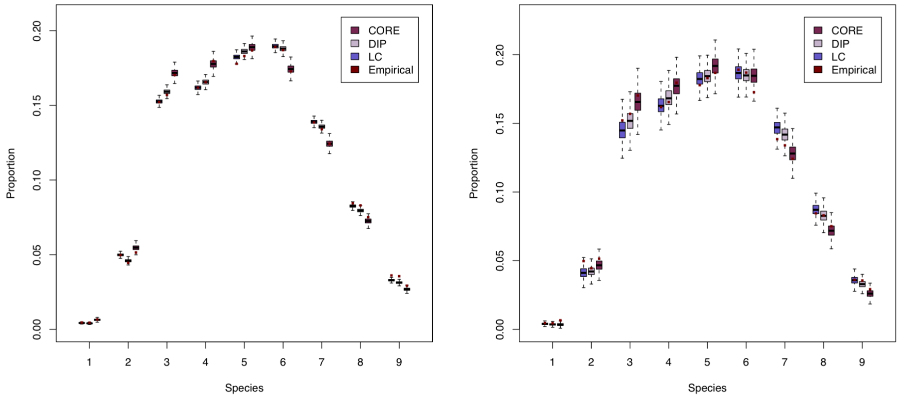

(a) *Network Shuffle* **[complex]**

(b) *Node Shuffle* **[complex]**

Figure S4: **Matching orthologues for each topology comparison**

Figure S5 - Similarity of protein trees according to their degree

This graph shows the level of similarity found for the protein trees when binned according to their degree. It is clear from the similarity results that the hub proteins play a significant role in the similarity of the topologies, but not clear what traits those few hubs actually have. There are no significant differences in the topological similarity of the trees by their degree.

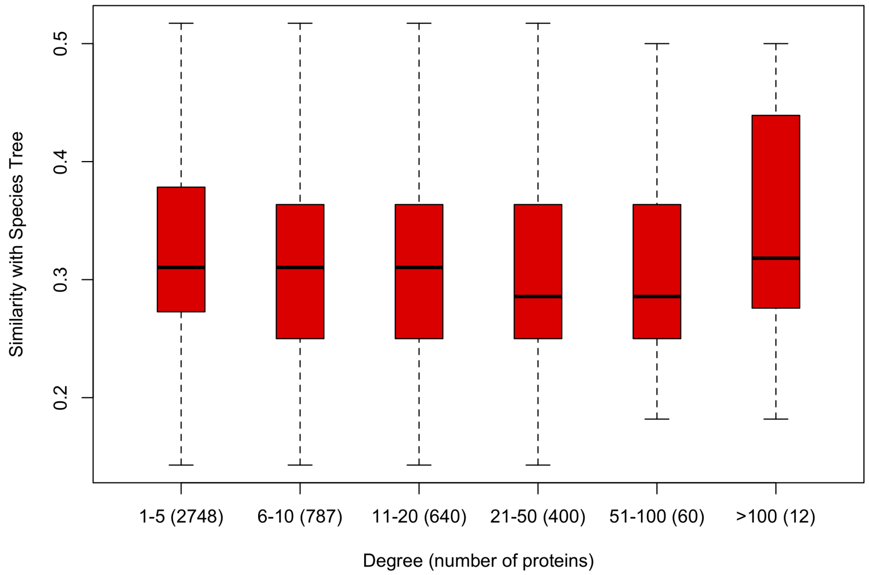

Figure S5: **Similarity of protein trees according to their degree**
